# HNRNPA2B1 as an emerging coordinator of RNA fate in cancer: m6A reading, RNA export, translation reprogramming, and immune–metabolic adaptation

**DOI:** 10.3389/fimmu.2026.1805984

**Published:** 2026-05-28

**Authors:** Wentao Bo, Biao Zhao, Yali Wang, Quan Dai

**Affiliations:** 1 Department of Hepatopancreatobiliary Surgery, Sichuan Clinical Research Center for Cancer, Sichuan Cancer Hospital and Institute, Sichuan Cancer Center, University of Electronic Science and Technology of China, Chengdu, China; 2 Department of Integrated Traditional Chinese and Western Medicine, Sichuan Clinical Research Center for Cancer, Sichuan Cancer Hospital and Institute, Affiliated Cancer Hospital of University of Electronic Science and Technology of China, Chengdu, China; 3 Department of Pharmacy, Beijing Anzhen Nanchong Hospital, Capital Medical University & Nanchong Central Hospital, Nanchong, China; 4 Department of Ultrasound, Center for Translational Research in Cancer, Sichuan Clinical Research Center for Cancer, Sichuan Cancer Hospital & Institute, Affiliated Cancer Hospital of University of Electronic Science and Technology of China, Chengdu, China

**Keywords:** HNRNPA2B1, immune–metabolic adaptation, m6A modification, RNA fate regulation, therapy resistance

## Abstract

Cancer progression and treatment failure are increasingly recognized as consequences of tumor plasticity rather than static genetic alterations. A key component of this plasticity is the ability of cancer cells to reprogram RNA fate, thereby reshaping gene expression outputs in response to microenvironmental stress, immune surveillance, infection, and therapeutic pressure. Among RNA-binding proteins involved in post-transcriptional regulation, heterogeneous nuclear ribonucleoprotein A2/B1 (HNRNPA2B1) has been increasingly implicated in several cancer-associated RNA regulatory processes that extend beyond its initial characterization as an N6-methyladenosine (m6A) reader. In this review, we synthesize recent studies showing that HNRNPA2B1 participates in multiple aspects of RNA regulation, including m6A-dependent stabilization of oncogenic lncRNAs and mRNAs, ISGylation-associated selective nuclear export of m6A-tagged transcripts, cytoplasmic translation control, and extracellular vesicle–mediated RNA communication. We further discuss studies associating HNRNPA2B1 with immune–metabolic phenotypes and therapy response, including tumor acidosis, ferroptosis-related pathways, immune evasion, and altered sensitivity to chemotherapy, radiotherapy, endocrine therapy, and targeted treatments. Importantly, these mechanisms have largely been described in distinct tumor types, experimental systems, and biological contexts, and therefore should not yet be interpreted as a single universally established regulatory cascade. Instead, we organize current evidence within a context-aware framework in which HNRNPA2B1 is discussed as a possible regulator of selected RNA fate modules rather than as an established pan-cancer RNA fate hub. By distinguishing experimentally supported mechanisms from hypothesis-generating interpretations, this review provides a balanced assessment of the current evidence, limitations, and therapeutic implications of HNRNPA2B1-related RNA regulation in cancer.

## Highlights

- HNRNPA2B1 is an emerging context-dependent regulator of selected RNA fate processes in cancer.
- Current evidence supports several HNRNPA2B1-related functional modules rather than a single unified RNA fate cascade.
- Further integrated studies are needed to define the mechanistic hierarchy and therapeutic relevance of HNRNPA2B1-associated RNA regulation.

## 1 Introduction

Cancer progression is increasingly recognized as a process driven not only by genetic alterations but also by the remarkable plasticity of gene expression programs that allow tumor cells to adapt to fluctuating microenvironmental and therapeutic pressures. While transcriptional regulation has long been considered the dominant layer of gene control, it has become evident that post-transcriptional regulation of RNA fate constitutes an equally critical, and often more rapidly adjustable, determinant of malignant behavior. The concept of RNA fate encompasses a series of tightly coordinated processes that collectively determine the functional output of RNA molecules, including RNA stability, alternative splicing, nuclear export, translation efficiency, and intercellular transport via extracellular vesicles (1–6). Rather than acting as isolated steps, these processes form an integrated regulatory continuum that shapes when, where, and how RNA molecules exert their biological effects. Subtle alterations at any stage of this continuum can profoundly reprogram cellular phenotypes without requiring changes at the DNA level.

In cancer, dysregulation of RNA fate enables tumor cells to rapidly remodel their proteome in response to environmental stressors such as nutrient limitation, hypoxia, immune surveillance, infection, and anticancer therapies. For instance, enhanced RNA stability can sustain oncogenic signaling despite transcriptional repression, while selective translation of stress-responsive transcripts allows tumor cells to survive cytotoxic insults (7–11). Similarly, altered RNA sorting into extracellular vesicles facilitates intercellular communication that reshapes the tumor microenvironment and promotes metastatic niche formation. Importantly, tumor adaptability is rarely governed by a single signaling pathway. Instead, it emerges from system-level reprogramming of RNA fate, in which multiple RNA processing steps are coordinately adjusted to support malignant plasticity. This realization has shifted attention toward RNA-binding proteins (RBPs) as central regulators of cancer biology, given their unique capacity to integrate diverse upstream signals and impose selective control over RNA populations (12, 13).

Among the various layers of post-transcriptional regulation, N6-methyladenosine (m6A) modification has emerged as the most prevalent and dynamically regulated internal modification of eukaryotic mRNA and non-coding RNA. m6A influences nearly every aspect of RNA metabolism, including RNA stability, splicing, nuclear export, translation, and decay, thereby serving as a versatile epitranscriptomic mark that fine-tunes gene expression programs (14, 15). The functional consequences of m6A are executed through a coordinated writer–eraser–reader system. m6A writers catalyze the deposition of the modification, erasers remove it in a reversible manner, and readers interpret the m6A signal to direct downstream RNA fate decisions. Dysregulation of any component of this system has been implicated in tumor initiation, progression, and therapeutic resistance across multiple cancer types. While much attention has focused on m6A writers and erasers, accumulating evidence highlights RNA-binding proteins that function as m6A readers as particularly critical determinants of context-specific RNA regulation (16–21). Unlike enzymatic regulators, readers do not simply modify RNA but instead translate epitranscriptomic information into functional outcomes, often in a cell type–, stimulus-, or subcellular localization–dependent manner. HNRNPA2B1 was initially characterized as a predominantly nuclear RNA-binding protein involved in RNA processing and transport and later identified as an m6A reader capable of recognizing methylated RNA motifs. Early studies positioned HNRNPA2B1 as a mediator linking m6A modification to RNA splicing and stability. However, emerging evidence over the past five years has substantially expanded this view, revealing that HNRNPA2B1 operates far beyond a passive m6A recognition factor. Recent studies demonstrate that HNRNPA2B1 exhibits remarkable functional versatility, engaging in both m6A-dependent and m6A-independent regulatory programs, dynamically shuttling between nuclear and cytoplasmic compartments, and interacting with diverse protein complexes involved in RNA export, translation initiation, and extracellular vesicle cargo selection. These findings suggest that HNRNPA2B1 should not be viewed solely as an m6A reader, but may participate in several context-dependent RNA regulatory processes that influence cancer cell adaptation.

Based on accumulating evidence from diverse tumor models, HNRNPA2B1 appears to participate in multiple dimensions of post-transcriptional regulation, including m6A-dependent and m6A-independent control of RNA stability, nuclear export, translation, and intercellular communication (22–26). However, these functions have largely been described in distinct cancer types, experimental systems, and biological contexts, and therefore should not yet be interpreted as a universally unified regulatory cascade. In this review, we organize these findings within a context-aware framework in which HNRNPA2B1 is discussed as an emerging regulator of selected RNA fate processes, with its reported functions shaped by cellular context, subcellular localization, RNA substrates, interacting partners, and upstream stimuli such as inflammation, metabolic stress, infection, and therapeutic pressure. By placing HNRNPA2B1 within the broader landscape of RNA fate regulation, we aim to evaluate the current evidence without implying that the reported mechanisms form a single, linear, or universally conserved RNA fate cascade.

## 2 Molecular basis of HNRNPA2B1 as an RNA fate hub

### 2.1 Structural features and RNA-binding properties

HNRNPA2B1 belongs to the heterogeneous nuclear ribonucleoprotein (hnRNP) family, a class of RNA-binding proteins characterized by their ability to associate with nascent and mature RNA transcripts and regulate multiple aspects of RNA metabolism. Structurally, HNRNPA2B1 contains two conserved RNA recognition motif (RRM) domains located in its N-terminal region, which constitute the primary interfaces for RNA binding (27–30). These RRMs confer both sequence preference and structural flexibility, enabling HNRNPA2B1 to engage with a wide spectrum of RNA substrates.

Biochemical and transcriptome-wide analyses have demonstrated that HNRNPA2B1 exhibits broad RNA-binding capacity, interacting not only with protein-coding mRNAs but also with long non-coding RNAs (lncRNAs) and circular RNAs (circRNAs) (31, 32). This promiscuous yet selective binding behavior reflects the intrinsic adaptability of its RRM domains, which can recognize diverse RNA motifs and secondary structures rather than rigid consensus sequences. Such binding versatility provides the molecular foundation for HNRNPA2B1 to function across distinct RNA regulatory contexts.

Importantly, HNRNPA2B1 has been identified as a reader of N6-methyladenosine (m6A), recognizing methylated RNA regions and translating epitranscriptomic marks into downstream regulatory outcomes (33, 34). Unlike some m6A readers that exert relatively uniform effects on RNA fate, HNRNPA2B1 appears to display context-dependent m6A recognition, whereby m6A marks enhance its affinity for specific transcripts without dictating a single functional consequence. This feature allows HNRNPA2B1 to selectively stabilize certain RNAs, promote their processing or export, or facilitate their engagement with additional regulatory complexes.

The ability of HNRNPA2B1 to interact with lncRNAs, circRNAs, and mRNAs suggests that it may serve as a flexible scaffold within selected RNA regulatory networks. In several cancer contexts, lncRNAs and circRNAs have been shown to recruit HNRNPA2B1 to specific mRNA targets, thereby conferring transcript selectivity and reinforcing oncogenic gene expression programs (35). Collectively, these structural and binding properties provide a molecular basis for the context-dependent involvement of HNRNPA2B1 in selected RNA fate decisions.

### 2.2 Dynamic subcellular localization

HNRNPA2B1 is predominantly nuclear under basal conditions, where it participates in RNA processing and export-related events (36, 37). However, stimulus-dependent cytoplasmic accumulation has been reported in specific contexts, including infection-associated gastric cancer models. This localization shift may enable HNRNPA2B1 to engage translation-related machinery, but it should not be interpreted as a universal transition from nuclear RNA processing to cytoplasmic translation.

### 2.3 Post-translational modifications fine-tune HNRNPA2B1 activity

Post-translational modifications may influence HNRNPA2B1 function by changing its binding partners, localization, or RNA regulatory output. ISGylation has been linked to selective mRNA export in defined settings, whereas the functional relevance of other modifications remains incompletely characterized (22, 24). These observations support a context-dependent view of HNRNPA2B1 activity rather than a single consolidated regulatory mechanism.

## 3 m6A-dependent RNA stabilization by HNRNPA2B1: evidence from selected cancer models

As an m6A reader, HNRNPA2B1 has been reported to translate epitranscriptomic signals into RNA fate outcomes in specific biological contexts. Among its m6A-dependent activities, selective stabilization of oncogenic RNAs has emerged as a recurrent and conceptual framework across multiple cancer types. Rather than globally enhancing RNA stability, HNRNPA2B1 preferentially protects specific non-coding and coding transcripts that are central to malignant phenotypes, thereby reinforcing tumor-promoting gene expression programs.

### 3.1 Stabilization of lncRNAs in cancer progression and drug resistance

**Figure 1:**
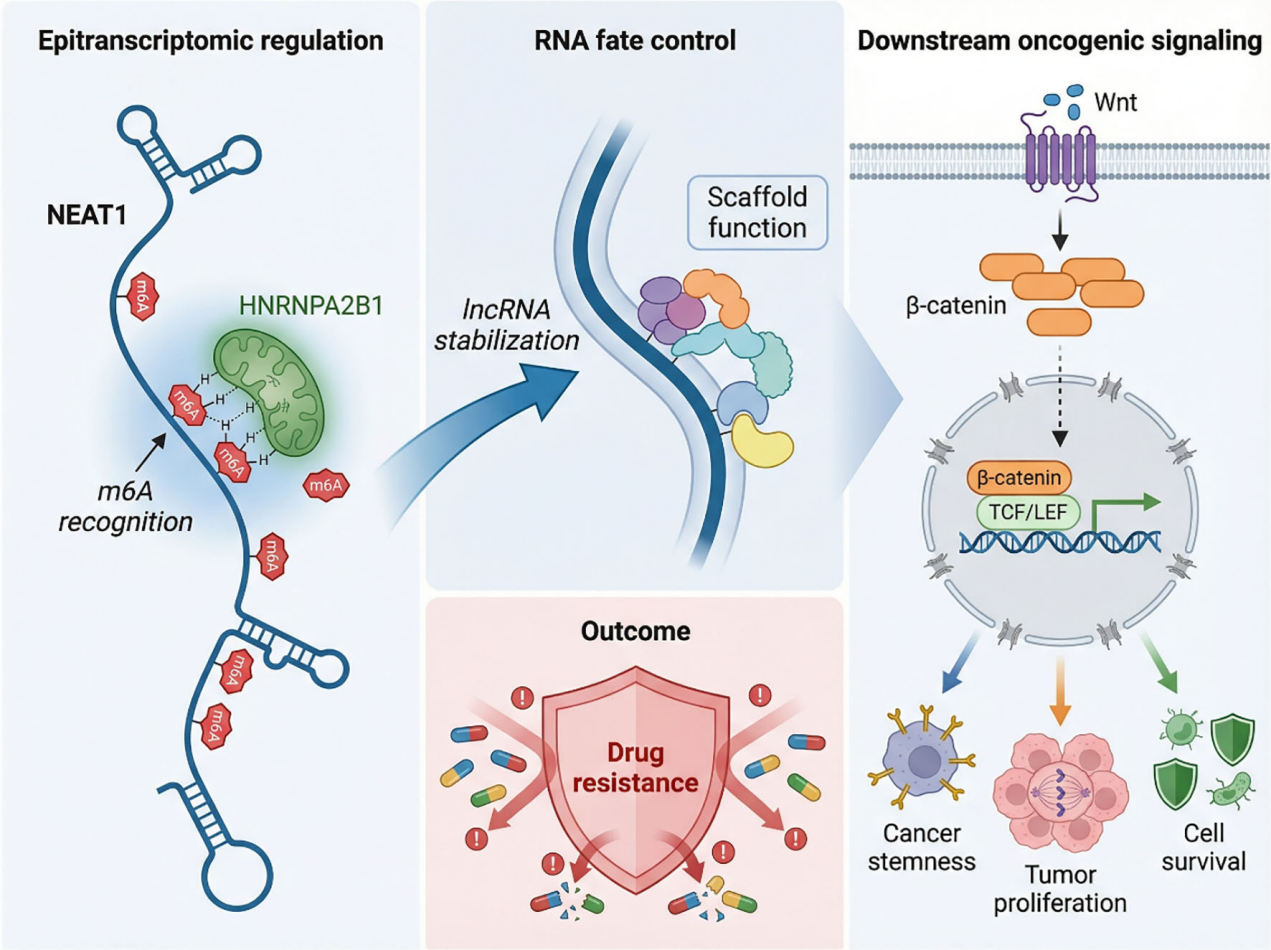
m6A-dependent lncRNA stabilization by HNRNPA2B1 in cancer progression and drug resistance. In gastric cancer, HNRNPA2B1 selectively recognizes m6A-modified NEAT1 and enhances its RNA stability. Stabilized NEAT1 acts as a molecular scaffold to reinforce Wnt/β-catenin signaling, thereby sustaining cancer stem–like programs, promoting tumor cell survival and proliferation, and ultimately conferring resistance to chemotherapeutic stress.

Long non-coding RNAs (lncRNAs) represent a major layer of regulatory complexity in cancer, functioning as scaffolds, decoys, and guides for RNA-binding proteins and chromatin modifiers. Increasing evidence indicates that m6A modification critically influences lncRNA stability and function, and HNRNPA2B1 has emerged as a key mediator linking m6A-marked lncRNAs to oncogenic outcomes (38–42). A representative and mechanistically well-defined example is the NEAT1–HNRNPA2B1 axis in gastric cancer. In this context, HNRNPA2B1 directly recognizes m6A-modified NEAT1 transcripts and enhances their stability, thereby sustaining high NEAT1 expression levels in tumor cells (43, 44). Stabilized NEAT1, in turn, promotes activation of the Wnt/β-catenin signaling pathway, a central driver of tumor stemness, proliferation, and survival (45–47). Through this cascade, HNRNPA2B1-mediated NEAT1 stabilization contributes to the maintenance of cancer stem–like properties and confers resistance to chemotherapeutic agents. Importantly, this mechanism illustrates a broader principle in RNA fate regulation: lncRNAs can function as molecular scaffolds that anchor HNRNPA2B1 to specific oncogenic programs. By binding to m6A-modified lncRNAs, HNRNPA2B1 is spatially and functionally positioned within defined regulatory circuits, enabling selective reinforcement of pathways associated with stemness, survival, and therapy resistance. This scaffold-based mode of action provides specificity to HNRNPA2B1 activity and prevents indiscriminate stabilization of the transcriptome. Beyond NEAT1, similar paradigms are likely operative in other cogenic signalinancer contexts, where m6A-modified lncRNAs recruit HNRNPA2B1 to shape gene expression landscapes in a context-dependent manner. Thus, lncRNA stabilization represents one experimentally supported route through which HNRNPA2B1 may influence cancer cell plasticity and drug responsiveness in selected contexts (**Figure 1**).

### 3.2 m6A-dependent stabilization of oncogenic mRNAs

In addition to its effects on non-coding RNAs, HNRNPA2B1 has been implicated in cancer biology through m6A-dependent stabilization of selected oncogenic mRNAs. Across multiple tumor types, HNRNPA2B1 selectively binds to m6A-marked transcripts encoding key signaling molecules, transcriptional regulators, and metabolic enzymes, thereby sustaining their expression and reinforcing malignant phenotypes (48).

In multiple myeloma, HNRNPA2B1 has been shown to stabilize m6A-modified mRNAs involved in pro-survival signaling. A notable example is the stabilization of transcripts within the ILF3–AKT3 axis, which promotes tumor cell proliferation and survival (49). By enhancing the half-life of these mRNAs, HNRNPA2B1 ensures persistent activation of AKT signaling, a pathway that is central to myeloma pathogenesis and therapy resistance (50).

Similarly, in colorectal cancer, HNRNPA2B1-mediated m6A recognition stabilizes oncogenic mRNAs such as TCF7L2, a key transcriptional effector of Wnt signaling (51, 52). Sustained TCF7L2 expression reinforces Wnt-driven transcriptional programs that support tumor growth, invasion, and stemness. In this setting, HNRNPA2B1 acts as a molecular bridge between epitranscriptomic modification and transcriptional network maintenance.

Evidence from breast cancer, prostate cancer, and other solid tumors further supports a conserved role for HNRNPA2B1 in stabilizing m6A-modified mRNAs that govern proliferation, metabolism, and therapy response (53). Notably, these targets vary across cancer types, underscoring the context-specific nature of HNRNPA2B1 function. Rather than stabilizing all m6A-marked transcripts, HNRNPA2B1 displays selective affinity for a subset of oncogenic mRNAs, reflecting the influence of RNA sequence features, structural elements, co-binding partners, and cellular context.

**Figure 2:**
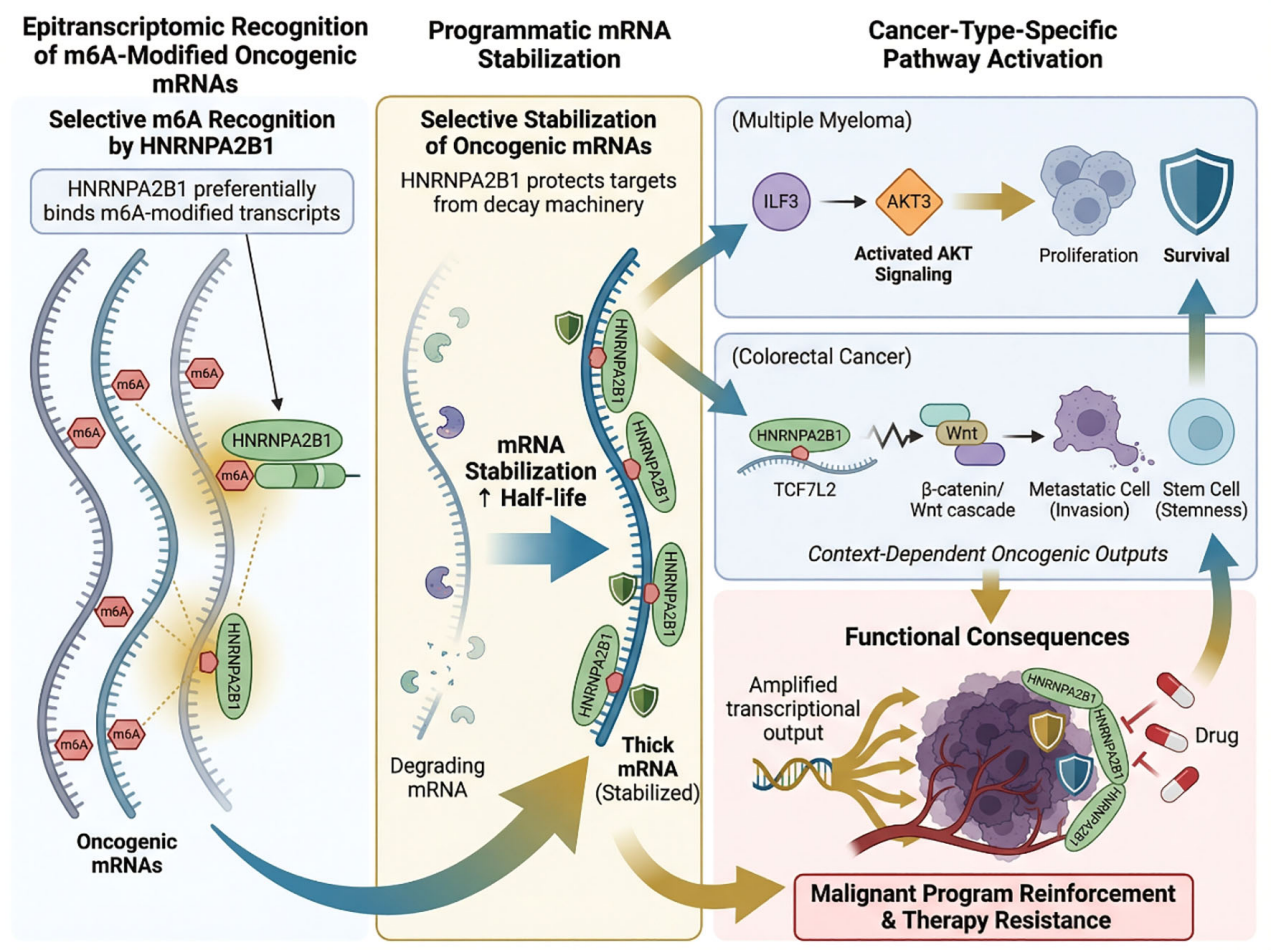
m6A-dependent stabilization of oncogenic mRNAs by HNRNPA2B1 in cancer. HNRNPA2B1 selectively recognizes m6A-modified oncogenic mRNAs and enhances their stability in a context-dependent manner. In multiple myeloma, stabilization of ILF3–AKT3 axis transcripts sustains AKT signaling to promote proliferation and survival. In colorectal cancer, HNRNPA2B1 stabilizes TCF7L2 mRNA, reinforcing Wnt-driven transcriptional programs that support tumor growth, stemness, and therapy resistance.

Collectively, these findings suggest that HNRNPA2B1-mediated m6A-dependent RNA stabilization can be selective rather than global, although the determinants of this selectivity remain incompletely understood. By preferentially stabilizing transcripts that converge on core oncogenic pathways, HNRNPA2B1 amplifies malignant gene expression programs while preserving overall transcriptomic balance. This selective stabilization provides a possible mechanism by which HNRNPA2B1 may influence cancer-associated gene expression programs in specific settings (**Figure 2**).

Taken together, m6A-dependent RNA stabilization represents a foundational mechanism through which HNRNPA2B1 regulates cancer cell behavior (54). By selectively stabilizing m6A-modified lncRNAs and mRNAs, HNRNPA2B1 reinforces oncogenic signaling networks, promotes tumor stemness, and facilitates drug resistance. Importantly, the specificity of this regulation is achieved through scaffold-based interactions and context-dependent target selection, positioning HNRNPA2B1 as a precision regulator of oncogenic RNA programs rather than a global RNA stabilizer. Although these studies support a role for HNRNPA2B1 in RNA stabilization, they do not by themselves establish a continuous regulatory sequence linking stabilization to export, translation, and extracellular vesicle sorting within the same biological system. Accordingly, the following sections should be interpreted as describing additional context-dependent dimensions of HNRNPA2B1 function rather than obligatorily sequential steps. The studies discussed in this section support HNRNPA2B1-mediated RNA stabilization in selected contexts, but they do not establish that stabilization is obligatorily linked to nuclear export, translation regulation, or extracellular vesicle sorting.

## 4 Context-specific selective nuclear export of m6A-tagged mRNAs

Recent work expands the functional scope of HNRNPA2B1 beyond RNA stability and suggests that, in specific contexts, it can also participate in selective mRNA export. Importantly, this export-related function has been demonstrated in defined settings and should not be assumed to operate uniformly across cancers. While m6A-dependent RNA stabilization represents a foundational mechanism of HNRNPA2B1-mediated gene regulation, recent evidence reveals that the regulatory scope of HNRNPA2B1 extends beyond RNA abundance to encompass spatial control of RNA fate (55). In particular, selective nuclear export of m6A-tagged mRNAs has emerged as a critical and previously underappreciated dimension of HNRNPA2B1 function. This process enables HNRNPA2B1 to couple epitranscriptomic recognition with RNA trafficking, thereby exerting control over gene expression at multiple sequential stages. Selective nuclear export is therefore discussed here as a distinct functional module rather than as a necessary downstream consequence of HNRNPA2B1-mediated RNA stabilization.

### 4.1 ISGylation-dependent regulation of HNRNPA2B1

**Figure 3:**
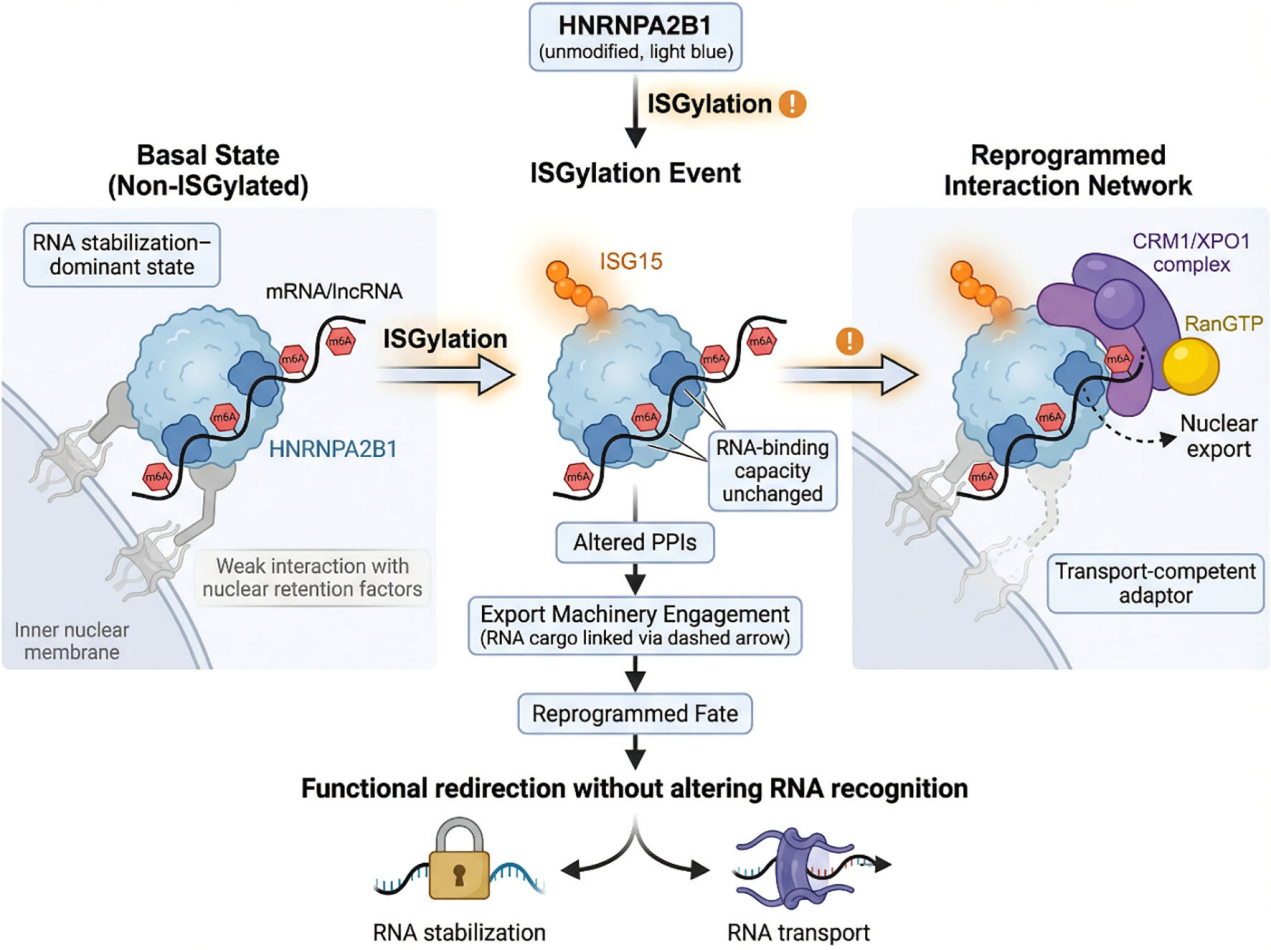
ISGylation-dependent functional reprogramming of HNRNPA2B1. ISGylation modifies HNRNPA2B1 without altering its RNA recognition capacity but reshapes its protein–protein interaction landscape. Through ISG15 conjugation, HNRNPA2B1 shifts from a predominantly RNA-stabilizing factor to a transport-competent adaptor, preferentially associating with nuclear export machinery and linking selected RNA substrates to downstream export pathways in a context-dependent manner.

Post-translational modification of RNA-binding proteins provides a powerful mechanism for dynamically reshaping RNA regulatory networks. In the case of HNRNPA2B1, ISGylation has been identified in specific settings as a modification that may fine-tune HNRNPA2B1 functional output. ISGylation alters the protein–protein interaction landscape of HNRNPA2B1, enabling it to engage with distinct regulatory complexes involved in RNA transport (56, 57). Mechanistically, ISGylation does not simply modulate the intrinsic RNA-binding affinity of HNRNPA2B1; rather, it reprograms its interaction repertoire, favoring associations with components of the nuclear export machinery. Through this modification, HNRNPA2B1 may acquire adaptor-like properties that link selected RNA substrates to downstream export pathways in defined experimental contexts. This regulatory mode highlights an important conceptual advance: post-translational modifications can redirect the functional trajectory of RNA-binding proteins without altering their RNA recognition capacity. These findings suggest that ISGylation may redirect HNRNPA2B1-associated RNA regulatory outcomes, although the breadth of this mechanism across tumor types remains to be clarified (**Figure 3**).

### 4.2 HNRNPA2B1–ALYREF/NXF1-mediated mRNA export and cancer-context evidence

**Figure 4:**
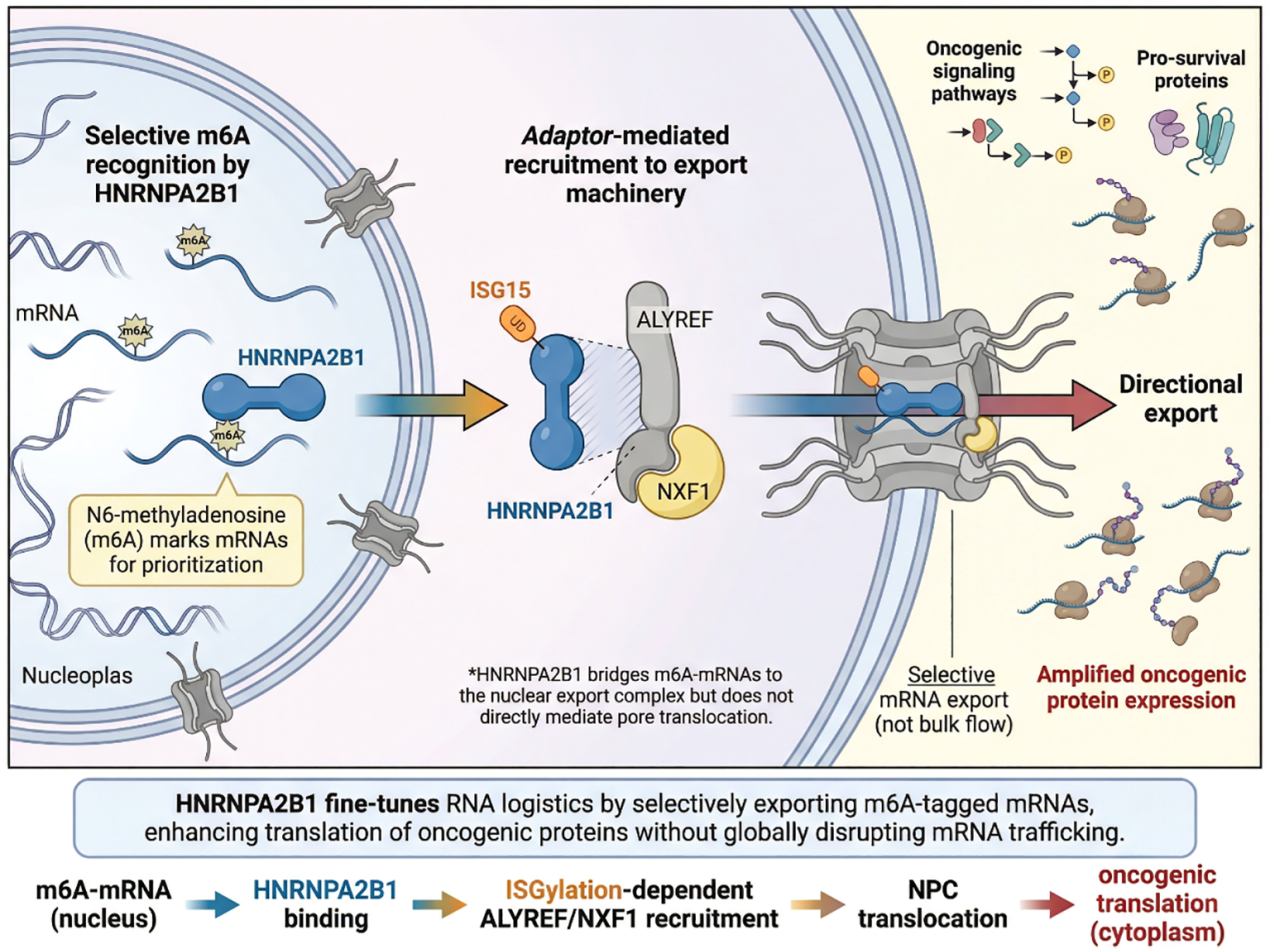
Selective mRNA nuclear export mediated by the HNRNPA2B1–ALYREF/NXF1 axis. Within the nucleus, HNRNPA2B1 recognizes m6A-tagged mRNAs and, upon ISGylation, functions as an adaptor linking selected transcripts to the ALYREF/NXF1 export complex. This interaction promotes prioritized nuclear export through the nuclear pore, ensuring efficient cytoplasmic delivery and translation of oncogenic and pro-survival mRNAs without globally perturbing bulk mRNA trafficking.

**Figure 5:**
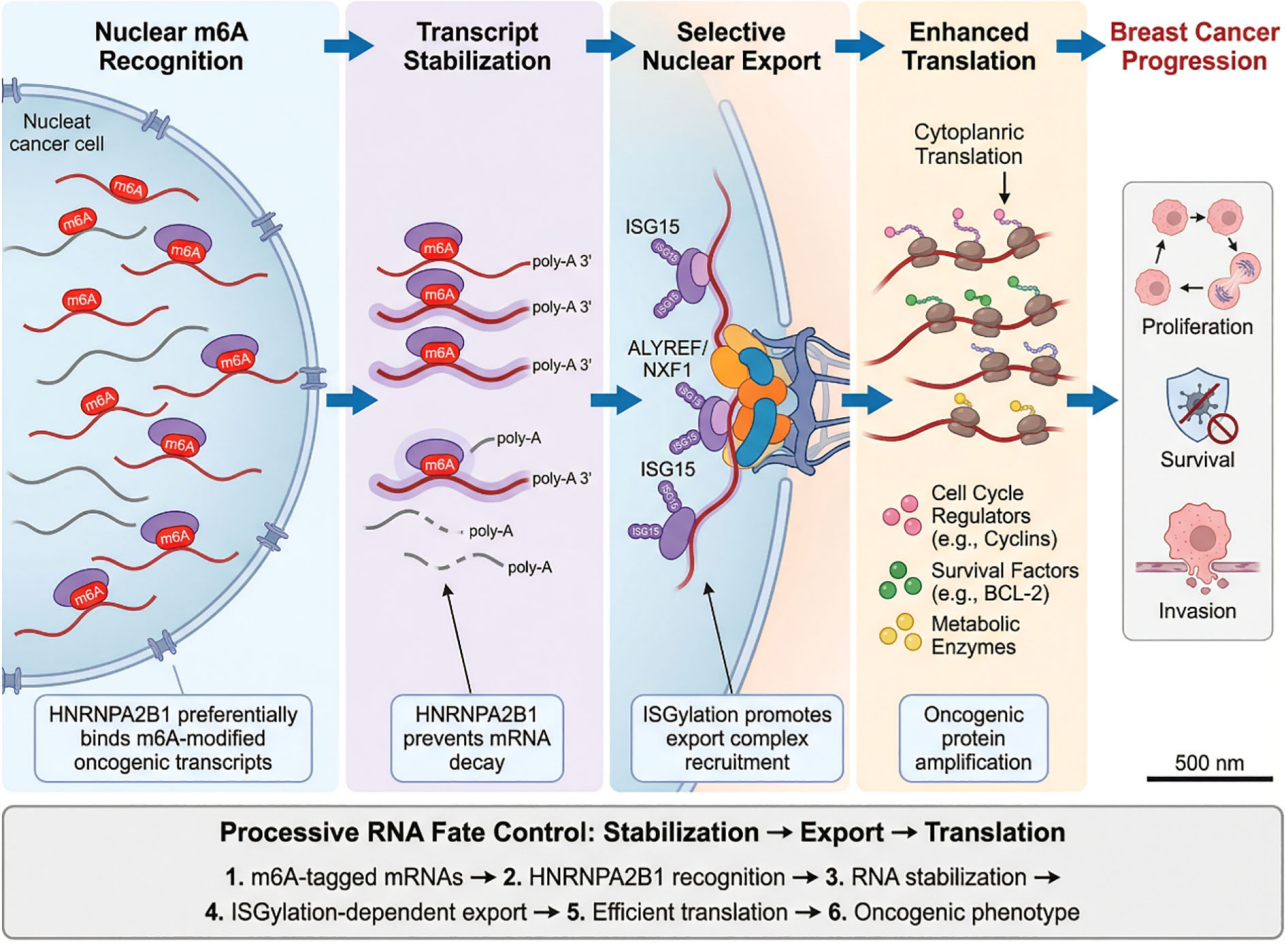
Processive RNA fate control by HNRNPA2B1 in breast cancer. In breast cancer cells, HNRNPA2B1 coordinates multiple stages of RNA regulation to amplify oncogenic protein output. By recognizing m6A-modified mRNAs, stabilizing selected transcripts, promoting their selective nuclear export, and enabling efficient cytoplasmic translation, HNRNPA2B1 establishes a continuous RNA fate pipeline that sustains proliferation, survival, metabolic activity, and aggressive tumor phenotypes.

Nuclear export of mRNA is a tightly regulated process that determines which transcripts gain access to the cytoplasmic translation machinery. The ALYREF/NXF1 export complex represents a major route for mature mRNA export from the nucleus to the cytoplasm (**Figure 4**) (37). In defined experimental settings, HNRNPA2B1 has been reported to function as an adaptor-like factor linking selected m6A-tagged transcripts to export-related machinery. In this model, HNRNPA2B1 recognizes specific m6A-modified mRNAs and, upon ISGylation, may interact more efficiently with components of the ALYREF/NXF1 export pathway, thereby facilitating the nuclear export of selected RNA substrates rather than globally enhancing bulk mRNA export. Evidence from breast cancer models provides a representative example of this export-related function. In these models, HNRNPA2B1-associated export of selected m6A-tagged oncogenic transcripts has been linked to increased cytoplasmic RNA availability and malignant phenotypes. However, these findings should be interpreted as cancer-context evidence for one export-related module, rather than proof that HNRNPA2B1 universally coordinates RNA stabilization, nuclear export, and translation as a continuous pathway across cancers. Whether HNRNPA2B1-mediated export is mechanistically coupled to upstream RNA stabilization or downstream translation control remains unresolved. Therefore, selective mRNA export should be viewed as a distinct and context-dependent functional module within the broader HNRNPA2B1 literature. Future studies combining subcellular RNA profiling, m6A mapping, HNRNPA2B1 perturbation, and export rescue experiments will be required to determine the specificity, cancer-type dependence, and functional hierarchy of this mechanism (**Figure 5**).

## 5 Context-specific m6A-independent translation regulation by cytoplasmic HNRNPA2B1

Distinct from its m6A-dependent nuclear functions, HNRNPA2B1 can also acquire a cytoplasmic, translation-related role under particular pathological conditions such as Helicobacter pylori infection. At present, this mechanism is best viewed as a context-specific functional mode rather than a general downstream continuation of all HNRNPA2B1-mediated RNA regulatory events. Although HNRNPA2B1 is widely recognized as an m6A reader that regulates RNA stability and nuclear export, emerging evidence reveals a distinct and highly context-dependent function of this protein in m6A-independent translation control. This non-canonical role expands the range of reported HNRNPA2B1 activities and suggests that, in specific contexts, it can function beyond canonical m6A interpretation. In particular, the reported ability of HNRNPA2B1 to influence translation independently of m6A modification supports its context-specific role as a translational regulator (58–61).

### 5.1 Helicobacter pylori–induced cytoplasmic relocalization of HNRNPA2B1

**Figure 6:**
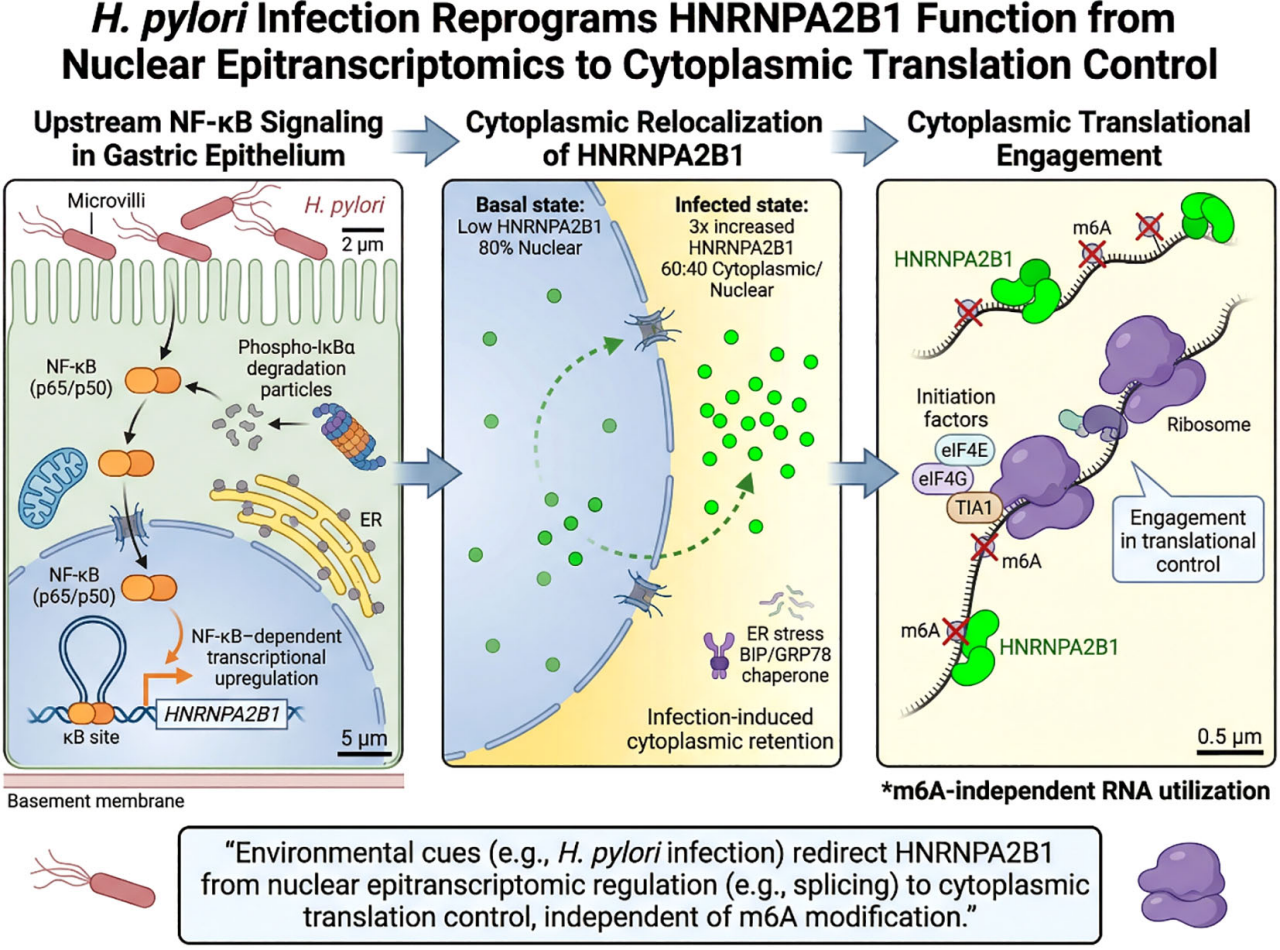
Helicobacter pylori–induced cytoplasmic relocalization of HNRNPA2B1 in gastric cancer. Chronic *H. pylori* infection activates NF-κB signaling, leading to transcriptional upregulation of HNRNPA2B1 and its infection-driven retention in the cytoplasm. This relocalization shifts HNRNPA2B1 from a nuclear RNA-processing factor to a cytoplasmic regulator with access to mRNA pools and translation initiation machinery, enabling functional reprogramming beyond its canonical m6A-dependent roles.

Chronic infection represents a potent driver of tumor evolution by reshaping host gene expression programs at multiple regulatory levels. In gastric cancer, Helicobacter pylori infection has been shown to induce a profound rewiring of RNA regulatory mechanisms, with HNRNPA2B1 emerging as a key effector of this process (62). Unlike its conventional nuclear localization, HNRNPA2B1 undergoes infection-induced relocalization to the cytoplasm, enabling it to participate directly in translational control. At the upstream level, *H. pylori* activates NF-κB signaling, which transcriptionally upregulates HNRNPA2B1 expression. Concomitantly, infection-associated signaling promotes the cytoplasmic retention of HNRNPA2B1, effectively shifting its functional emphasis away from nuclear RNA processing toward cytoplasmic RNA utilization (63, 64). This dual effect—enhanced expression coupled with altered subcellular localization—creates a permissive environment for HNRNPA2B1 to engage in translation-related functions. This relocalization appears to be functionally relevant rather than merely passive redistribution. By accumulating in the cytoplasm, HNRNPA2B1 gains access to translation initiation machinery and mRNA pools that are not primarily governed by m6A-dependent regulatory logic. Thus, infection-associated cytoplasmic accumulation of HNRNPA2B1 may represent a context-specific switch that allows the protein to operate beyond its canonical epitranscriptomic role (**Figure 6**).

### 5.2 Coordination with PABPC1 and the eIF4F complex

**Figure 7:**
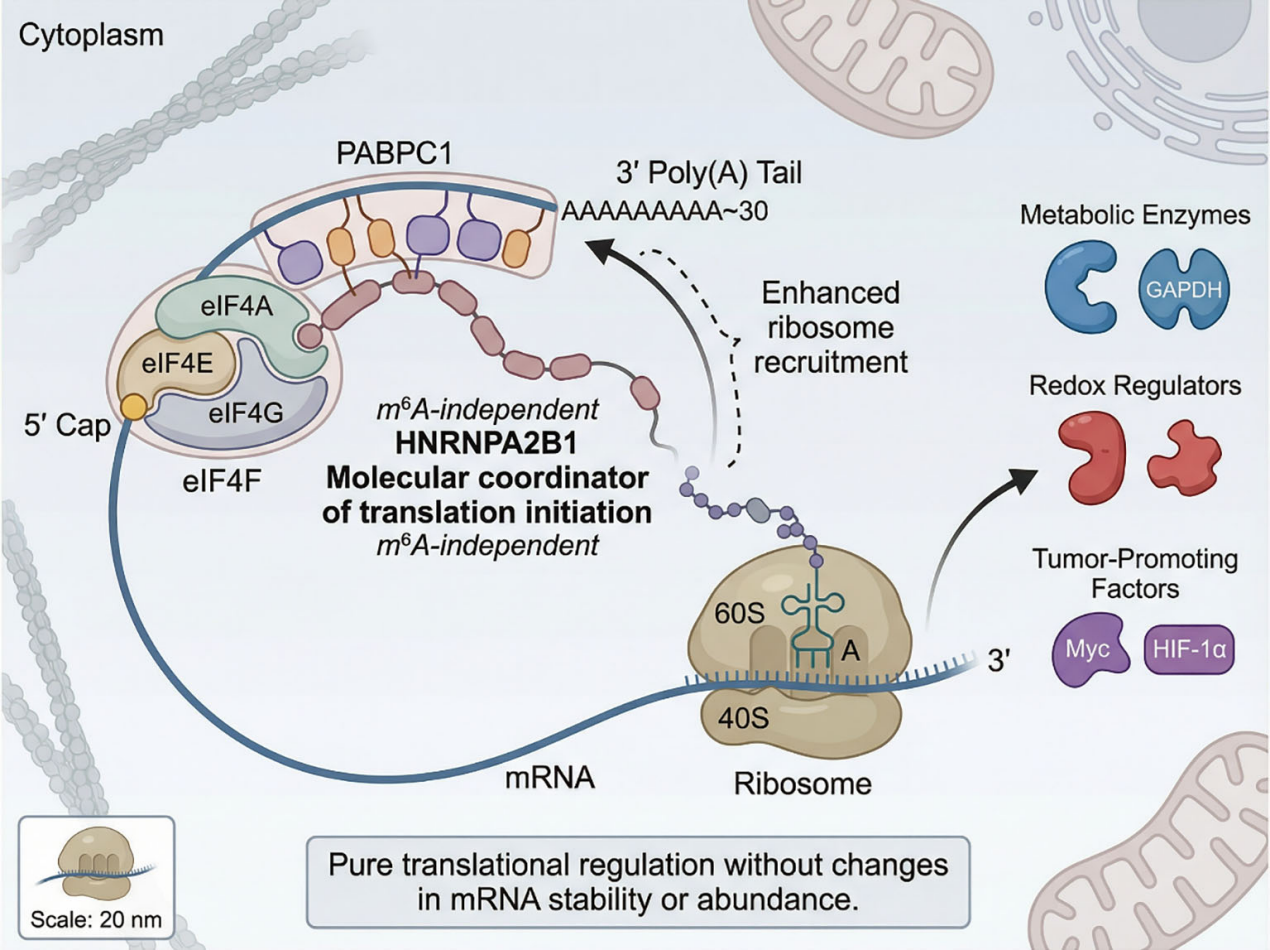
m6A-independent translational activation mediated by HNRNPA2B1 in cancer cells. Following cytoplasmic relocalization, HNRNPA2B1 coordinates with PABPC1 and the eIF4F complex to enhance cap-dependent translation initiation. By stabilizing the interaction between the poly(A)-binding protein and the translation initiation machinery, HNRNPA2B1 promotes efficient ribosome recruitment and selectively increases the synthesis of metabolic, redox, and tumor-promoting proteins without altering mRNA abundance or stability.

Once localized in the cytoplasm, HNRNPA2B1 exerts its translational regulatory function through direct coordination with the poly(A)-binding protein PABPC1 and the eIF4F translation initiation complex. This interaction positions HNRNPA2B1 at a pivotal checkpoint of protein synthesis: the recruitment of ribosomes to mRNA. Notably, the mRNAs regulated by cytoplasmic HNRNPA2B1 in this context are largely independent of m6A modification, marking a clear departure from its canonical reader function. Instead of recognizing methylated RNA motifs, HNRNPA2B1 facilitates translation by stabilizing the interaction between PABPC1 and eIF4F, thereby enhancing translation initiation efficiency. Through this mechanism, HNRNPA2B1 acts as a molecular coordinator that reinforces the physical and functional coupling between mRNA poly(A) tails and cap-dependent translation machinery. The translational targets of this pathway are functionally coherent rather than random. They are enriched for genes involved in metabolic regulation, redox homeostasis, and tumor-promoting activities, including enzymes and regulatory proteins that support rapid proliferation and stress tolerance (65–67). By selectively enhancing the translation of these transcripts, HNRNPA2B1 contributes to a metabolic and oxidative state that favors tumor survival and growth. Importantly, this mode of action does not require alterations in mRNA abundance or stability, highlighting a purely translational layer of regulation. Such m6A-independent translation reprogramming allows cancer cells to rapidly adjust protein output in response to environmental cues, bypassing slower transcriptional or epitranscriptomic mechanisms (**Figure 7**).

## 6 HNRNPA2B1-associated extracellular vesicle RNA sorting in specific tumor models

Although HNRNPA2B1 has been implicated in extracellular vesicle RNA sorting, the strength of evidence varies across experimental systems. Some studies provide direct support for HNRNPA2B1-dependent miRNA loading into EVs, whereas others infer its role from changes in EV RNA composition, metastatic phenotypes, or tumor–stroma communication. Therefore, EV-associated RNA sorting should be interpreted as a model-specific functional module rather than a universal downstream consequence of HNRNPA2B1 activity. A critical issue is whether HNRNPA2B1 directly selects specific RNA cargo, indirectly alters EV biogenesis or RNA abundance, or participates in both processes depending on cellular context. While the preceding sections emphasize the intracellular roles of HNRNPA2B1 in RNA stabilization, export, and translation, emerging evidence reveals an additional and conceptually transformative function of this protein in intercellular communication. Tumor progression is not solely determined by cell-autonomous processes; instead, it is critically shaped by dynamic interactions among cancer cells and diverse stromal and immune components within the tumor microenvironment. In this context, extracellular vesicles (EVs) have emerged as major vehicles for RNA-based communication, enabling the horizontal transfer of regulatory molecules between cells (68–70). HNRNPA2B1 has been implicated in RNA cargo selection into EVs in specific experimental models, suggesting that its influence on RNA fate may extend beyond the boundaries of individual cells. Through selective sorting of small RNAs into EVs, HNRNPA2B1 enables tumor cells to transmit regulatory information to neighboring or distant cells, thereby coordinating multicellular behaviors that support tumor growth and metastasis (71).

### 6.1 hnRNPA2B1-mediated miRNA sorting into extracellular vesicles

**Figure 8:**
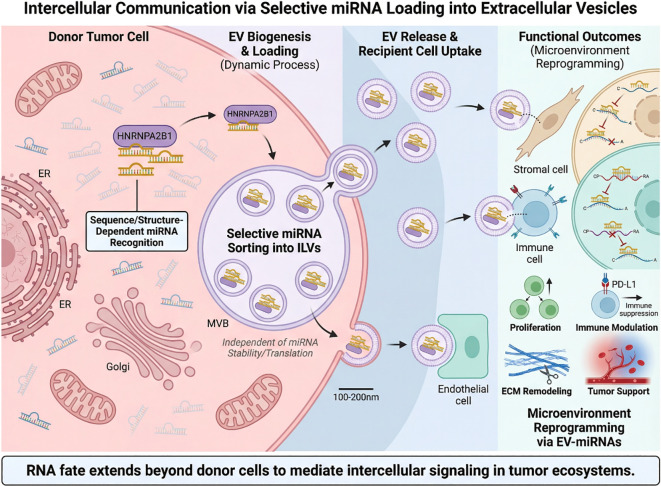
hnRNPA2B1-mediated selective miRNA sorting into extracellular vesicles. hnRNPA2B1 functions as an RNA selector that recognizes specific sequence or structural features in miRNAs and directs their preferential loading into extracellular vesicles. This selective sorting operates independently of intracellular miRNA stability or translation, ensuring EV-mediated delivery of tumor-promoting and microenvironment-modulating miRNAs to recipient cells, where they reprogram gene expression and support tumor progression.

The selective incorporation of microRNAs (miRNAs) into extracellular vesicles is a highly regulated process, rather than a passive reflection of intracellular RNA abundance. HNRNPA2B1 has been reported to participate in this process, functioning as an RNA-binding factor that may influence which miRNAs are preferentially packaged into EVs. Mechanistically, HNRNPA2B1 recognizes specific sequence or structural features within miRNAs and facilitates their enrichment in EVs. This selective sorting ensures that EVs carry a defined set of regulatory RNAs capable of exerting functional effects in recipient cells. By acting at this checkpoint, HNRNPA2B1 imposes an additional layer of RNA fate control that operates independently of RNA stability or translation within the donor cell. Importantly, miRNAs sorted into EVs under the guidance of HNRNPA2B1 are often enriched for tumor-promoting or microenvironment-modulating functions, including regulation of cell proliferation, differentiation, immune responses, and extracellular matrix remodeling (70, 72, 73). Once delivered to recipient cells, these miRNAs can reprogram gene expression profiles and alter cellular behavior in ways that favor tumor progression. This EV-based RNA selection mechanism highlights a critical expansion of the RNA fate concept: RNA destiny is not confined to intracellular outcomes but can include intentional export for intercellular signaling. Through hnRNPA2B1-mediated miRNA sorting, tumor cells actively sculpt the molecular messages they transmit to their surroundings (**Figure 8**). Mechanistically, the strongest evidence for HNRNPA2B1-mediated EV RNA sorting should ideally include several layers of validation: direct RNA binding, altered EV RNA cargo after HNRNPA2B1 perturbation, exclusion of changes in intracellular RNA abundance, rescue experiments using wild-type or RNA-binding-defective HNRNPA2B1, and functional assays in recipient cells. However, many cancer-related EV studies do not fully satisfy all these criteria. In some cases, reduced abundance of a specific EV miRNA after HNRNPA2B1 knockdown may reflect impaired cargo selection, but it may also result from altered miRNA transcription, processing, stability, or EV production. Therefore, claims of selective EV RNA sorting require careful experimental separation of cargo-loading effects from broader changes in RNA metabolism or vesicle biogenesis.

### 6.2 LSD1–HNRNPA2B1 axis in breast cancer bone metastasis

**Figure 9:**
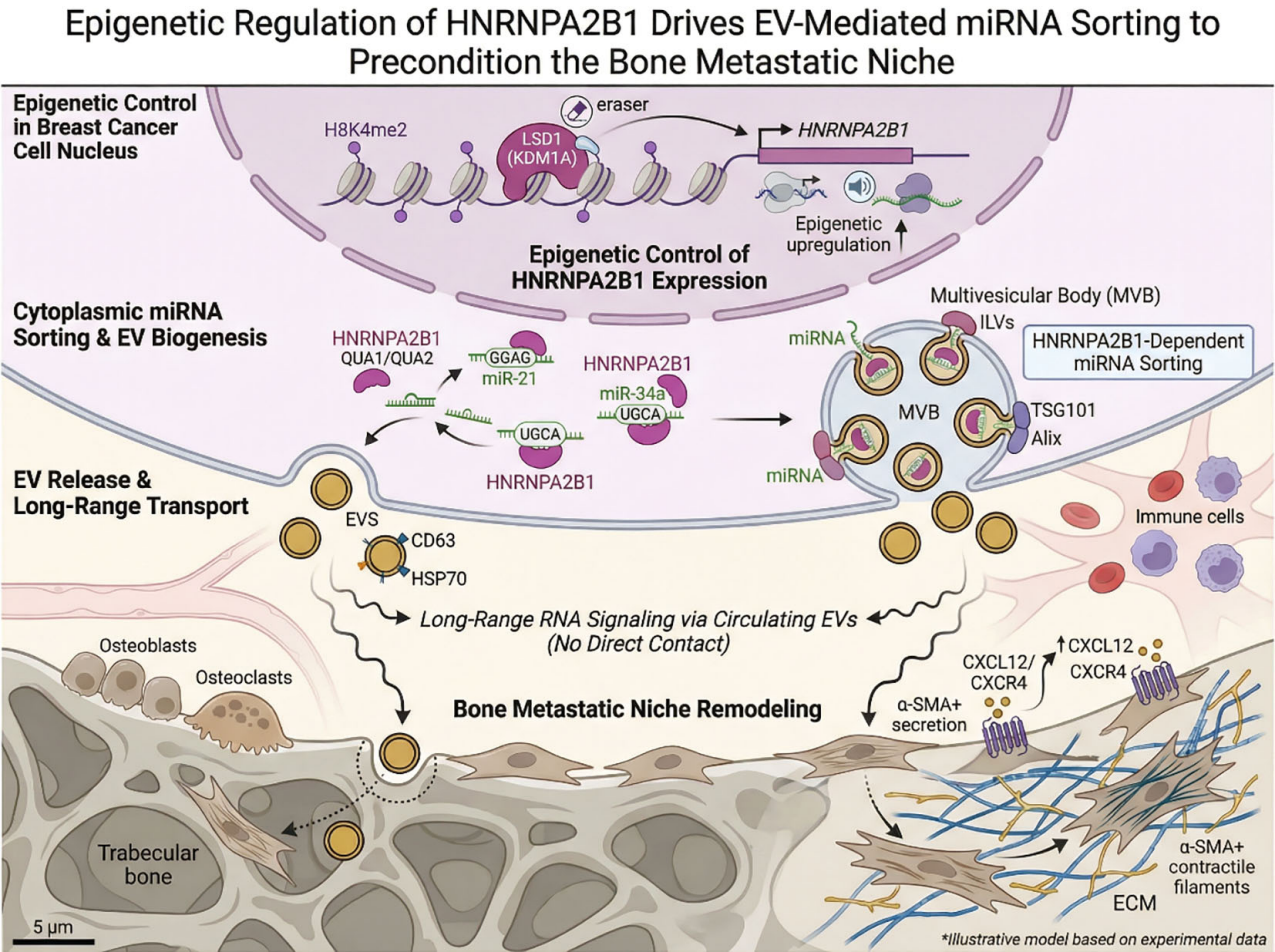
LSD1–HNRNPA2B1 axis drives EV-mediated niche remodeling in breast cancer bone metastasis. In breast cancer cells, the histone demethylase LSD1 regulates the expression and functional state of HNRNPA2B1, thereby shaping selective miRNA sorting into extracellular vesicles. These EV-associated miRNAs are delivered to stromal cells in the bone microenvironment, where they reprogram gene expression, promote myofibroblast activation and extracellular matrix remodeling, and establish a permissive metastatic niche.

A particularly illustrative example of HNRNPA2B1-driven intercellular communication is observed in breast cancer bone metastasis, where RNA cargo sorting into EVs plays a pivotal role in shaping the metastatic niche (74). In this setting, HNRNPA2B1 operates downstream of epigenetic regulation, forming a regulatory axis that links chromatin remodeling to extracellular RNA signaling. The histone demethylase LSD1 modulates the expression and functional state of HNRNPA2B1, thereby indirectly influencing EV-mediated RNA communication. Through this epigenetic–post-transcriptional coupling, LSD1 controls the repertoire of miRNAs that HNRNPA2B1 selects for EV packaging. These EV-associated miRNAs are subsequently delivered to stromal cells within the bone microenvironment, including fibroblasts and other mesenchymal cell populations. Upon uptake by recipient cells, the transferred miRNAs reprogram gene expression to promote myofibroblast activation and microenvironmental remodeling, processes that are essential for metastatic colonization and outgrowth. This mechanism underscores a sophisticated regulatory cascade: epigenetic modification in tumor cells is translated into post-transcriptional RNA selection, which is then externalized via EVs to rewire distant cellular ecosystems. Notably, this pathway exemplifies how HNRNPA2B1 enables tumor cells to exert long-range influence without direct cell–cell contact. By governing the RNA content of EVs, HNRNPA2B1 transforms EVs into targeted delivery systems for regulatory RNAs that precondition metastatic niches (**Figure 9**).

### 6.3 Current limitations and validation priorities

Several limitations currently restrict the interpretation of HNRNPA2B1-associated EV RNA sorting in cancer. First, EV isolation methods vary substantially across studies, and contamination by non-vesicular ribonucleoprotein complexes can confound RNA cargo analysis. Second, changes in EV RNA cargo are not always normalized to intracellular RNA abundance or total EV production, making it difficult to distinguish selective sorting from altered RNA availability or vesicle secretion. Third, many studies identify EV-associated RNAs but provide limited evidence that these RNAs are functionally delivered to recipient cells at biologically meaningful levels. Fourth, the relationship between HNRNPA2B1-mediated intracellular RNA regulation and EV RNA sorting remains unclear; these functions may be independent rather than sequential. Future studies should combine standardized EV isolation, quantitative EV characterization, HNRNPA2B1 perturbation, RNA-binding assays, EV RNA profiling, recipient-cell functional assays, and *in vivo* rescue experiments to determine whether HNRNPA2B1 directly drives EV-mediated tumor ecosystem remodeling.

## 7 HNRNPA2B1-associated immune–metabolic phenotypes in cancer

Tumor progression is increasingly understood as a process of continuous adaptation to immune surveillance and metabolic stress within the tumor microenvironment. Immune evasion, metabolic rewiring, and resistance to regulated cell death programs are not independent phenomena; rather, they are tightly interconnected processes that collectively determine tumor fitness. Emerging evidence suggests that HNRNPA2B1 may influence immune–metabolic phenotypes at the RNA level in selected tumor contexts, potentially contributing to cancer cell adaptation under immune and therapeutic pressure (75–77). Unlike classical oncogenic drivers that act primarily through signaling cascades or metabolic enzymes, HNRNPA2B1 exerts its influence by selectively reshaping RNA fate programs. Through these context-specific mechanisms, HNRNPA2B1-related RNA regulation may link metabolic outputs with immune-modulatory consequences, although direct causal connections remain incompletely defined.

### 7.1 Lactate accumulation, acidosis, and immune escape

Metabolic reprogramming is a hallmark of cancer and a major determinant of immune responsiveness within the tumor microenvironment. In non-small cell lung cancer (NSCLC), HNRNPA2B1 has been shown to promote a metabolic state characterized by enhanced lactate production and extracellular acidification (78, 79). This metabolic shift is not merely a byproduct of increased glycolysis but represents an adaptive strategy that directly undermines antitumor immunity. By regulating RNA programs associated with glycolytic flux and lactate accumulation, HNRNPA2B1 may contribute to the establishment of an acidic tumor microenvironment in selected models (80). Tumor acidosis exerts profound immunosuppressive effects, particularly on CD8^+^ cytotoxic T lymphocytes, impairing their proliferation, cytokine production, and effector function. As a result, immune surveillance is weakened, allowing tumor cells to evade immune-mediated elimination. In parallel, lactate-rich and acidic conditions have been linked to suppression of ferroptosis, a form of iron-dependent regulated cell death that is sensitive to redox imbalance. Through RNA-associated metabolic changes, HNRNPA2B1 may indirectly influence ferroptosis sensitivity, although this relationship requires further mechanistic validation. These findings highlight a critical insight: HNRNPA2B1-mediated RNA regulation can shape metabolic outputs that simultaneously promote immune escape and cell death resistance.

### 7.2 Ferroptosis and redox homeostasis

Beyond its effects on metabolic acidification, HNRNPA2B1 plays a direct role in maintaining redox homeostasis and ferroptosis resistance, particularly in the context of therapeutic stress (81, 82). Ferroptosis is increasingly recognized as a key vulnerability in cancer cells, especially under conditions of radiotherapy or oxidative stress. However, cancer cells frequently acquire mechanisms to suppress ferroptotic signaling and preserve redox balance.

In NSCLC, HNRNPA2B1 has been implicated in autophagy-dependent regulation of ferroptosis, where it modulates RNA programs that influence iron metabolism, lipid peroxidation, and antioxidant capacity. By selectively regulating transcripts involved in these pathways, HNRNPA2B1 helps maintain cellular redox equilibrium and prevents lethal lipid peroxidation. A compelling example of this regulatory axis is observed in the context of radiotherapy resistance. The HNRNPA2B1/HDGF/PTN axis has been shown to promote resistance to radiation-induced cell death by coordinating autophagic processes and ferroptosis suppression (83, 84). Through RNA-level control, HNRNPA2B1 ensures sustained expression of protective factors that buffer oxidative damage and enable tumor cells to withstand radiotherapy. These findings underscore a broader principle: RNA fate regulation by HNRNPA2B1 provides a flexible and rapid means to modulate ferroptosis sensitivity, allowing cancer cells to adapt to fluctuating redox conditions and therapeutic interventions.

### 7.3 Remodeling tumor–immune cell interactions

In addition to shaping metabolic and redox states, HNRNPA2B1 exerts profound effects on tumor–immune cell interactions, particularly in colorectal cancer (CRC) (39, 65, 85–87). Recent evidence demonstrates that HNRNPA2B1 orchestrates immune evasion by reprogramming RNA networks that govern immune cell recruitment and function within the tumor microenvironment. In CRC, elevated HNRNPA2B1 activity is associated with a marked reduction in CD8^+^ T-cell infiltration, a key determinant of poor prognosis and resistance to immunotherapy (88). Rather than acting only through direct immune checkpoint regulation, HNRNPA2B1 may influence the tumor–immune landscape by regulating RNA programs associated with cytokine signaling, chemokine gradients, and cell–cell communication. This RNA-centric remodeling of immune interactions leads to a reorganization of the tumor–immune cell network, favoring immunosuppressive niches and attenuated cytotoxic responses. Importantly, this mechanism integrates seamlessly with the metabolic and ferroptotic adaptations described above, as immune exclusion and metabolic suppression often reinforce one another within the tumor microenvironment. Collectively, these observations support a working model in which HNRNPA2B1 may contribute to immune evasion through several context-dependent RNA regulatory mechanisms. However, whether metabolic, redox, and immune-regulatory outputs are directly coordinated by HNRNPA2B1 within the same tumor system remains unresolved.

Taken together, current evidence suggests that HNRNPA2B1 may be associated with several immune–metabolic phenotypes in cancer, including lactate accumulation, extracellular acidification, altered ferroptosis sensitivity, and remodeling of tumor–immune interactions. However, these phenotypes have generally been described in different tumor types and experimental settings. Therefore, they should be interpreted as context-dependent consequences of HNRNPA2B1-associated RNA regulation rather than as components of a single unified immune–metabolic program. Importantly, whether metabolic remodeling, ferroptosis regulation, and immune evasion are directly coordinated by HNRNPA2B1 within the same tumor system remains to be determined.

## 8 HNRNPA2B1-associated RNA regulatory mechanisms in therapy resistance

Therapy resistance represents an important clinical phenotype associated with HNRNPA2B1-related RNA regulation. However, the available evidence is distributed across different cancer types, treatment modalities, and experimental systems. To avoid reiterating the mechanistic modules discussed above, this section summarizes therapy-resistance evidence according to treatment context rather than restating the underlying RNA regulatory mechanisms in detail. Overall, HNRNPA2B1 has been linked to chemotherapy resistance, radiotherapy resistance, endocrine therapy resistance, and altered sensitivity to targeted agents, but these associations should be interpreted as context-specific rather than as evidence for a universal resistance program. In gastric cancer, HNRNPA2B1-mediated stabilization of NEAT1 has been linked to Wnt/β-catenin activation, cancer stem–like properties, and chemotherapy resistance (36). In NSCLC, HNRNPA2B1-related regulation of autophagy and ferroptosis-associated pathways has been implicated in radiotherapy resistance (83). In breast cancer, HNRNPA2B1 has been associated with endocrine therapy resistance and altered PARP inhibitor response, although the precise RNA substrates and mechanistic hierarchy require further validation (89). These examples indicate that HNRNPA2B1 may contribute to therapy adaptation through distinct mechanisms in different treatment settings. Therefore, therapy resistance should be presented as a convergent phenotype of several context-dependent RNA regulatory modules, rather than as a single HNRNPA2B1-driven resistance pathway. To reduce conceptual repetition and clarify the strength of available evidence, we summarized HNRNPA2B1-associated therapy resistance according to treatment context and cancer type in **Table 1**. This summary avoids treating therapy resistance as a single unified mechanism and instead highlights the treatment- and cancer-type-specific nature of current evidence.

**Table 1:** HNRNPA2B1-associated therapy resistance across cancer contexts.

Treatment context	Cancer type/model	HNRNPA2B1-related mechanism	Main pathway or RNA substrate	Interpretation
Chemotherapy	Gastric cancer	m6A-dependent stabilization of lncRNA	NEAT1–Wnt/β-catenin	Direct context-specific evidence
Radiotherapy	NSCLC	Regulation of autophagy and ferroptosis-associated pathways	HNRNPA2B1/HDGF/PTN axis	Direct/context-specific evidence
Endocrine therapy	Breast cancer	RNA regulatory adaptation	ER-related survival programs	Association; mechanism requires clarification
PARP inhibitor response	Breast cancer	Regulation of RNA programs linked to DNA damage response and survival	DDR/autophagy-related pathways	Context-specific evidence
Targeted therapy	Selected tumor models	Stabilization or translation of adaptive transcripts	AKT/Wnt/metabolic pathways	Hypothesis-generating or model-dependent

## 9 Context specificity, heterogeneity, and current limitations

To clarify the strength and boundaries of current evidence, we summarized the major HNRNPA2B1-associated mechanisms according to their representative experimental support and interpretive status (**Table 2**). This evidence-level overview distinguishes mechanisms that have been directly validated in selected cancer models from broader phenotype-level associations or hypothesis-generating interpretations. Despite the expanding body of literature linking HNRNPA2B1 to multiple aspects of cancer biology, several important limitations should be considered when interpreting it as a regulator of RNA fate (22, 90). First, the currently available evidence is highly fragmented across tumor types, experimental systems, and biological contexts. Mechanisms such as m6A-dependent RNA stabilization, ISGylation-associated selective nuclear export, cytoplasmic translation control, extracellular vesicle cargo sorting, immune–metabolic adaptation, and therapy resistance have often been described in different cancers or under distinct stimuli. As a result, although these findings can be organized into a broader interpretive framework, they do not yet establish a single continuous or universally conserved regulatory cascade. Second, the functions of HNRNPA2B1 appear to be strongly context-dependent. In some settings, HNRNPA2B1 primarily operates as an m6A reader involved in RNA stabilization or processing, whereas in others it acquires non-canonical roles in translation regulation, extracellular RNA communication, or microenvironmental remodeling. These functional differences are likely shaped by multiple variables, including tumor lineage, cellular state, subcellular localization, binding partners, post-translational modifications, and the nature of upstream stress signals such as inflammation, infection, metabolic pressure, or therapeutic challenge. Therefore, it remains unclear whether m6A-dependent and m6A-independent mechanisms represent coordinated regulatory modes within the same cells or instead reflect distinct functional states observed under different pathological conditions (24). Third, the proposed relationship among RNA stabilization, nuclear export, translation regulation, and extracellular vesicle sorting should be interpreted with caution. In the current literature, these processes are often supported by separate mechanistic studies rather than by integrated analyses performed within the same biological system. Accordingly, the organization of these functions into an “RNA fate” framework is conceptually useful, but should not be mistaken for definitive evidence that HNRNPA2B1 imposes processive control across the entire RNA life cycle in a linear and unified manner. More direct studies will be required to determine whether these regulatory layers are mechanistically coupled in individual tumor contexts (37, 51). Fourth, much of the available evidence remains limited in scope. Many studies focus on single downstream targets, single pathways, or one tumor model, which makes it difficult to assess how generalizable the reported mechanisms are. In several cases, the apparent system-level role of HNRNPA2B1 is inferred from selected target transcripts or phenotypes rather than demonstrated through transcriptome-wide, multi-omic, or cross-context analyses. Future work integrating RNA interactome mapping, epitranscriptomic profiling, subcellular localization studies, and functional perturbation across matched tumor models will be important for clarifying the breadth and hierarchy of HNRNPA2B1-dependent regulation.

**Table 2:** Evidence levels of HNRNPA2B1-associated mechanisms in cancer.

Functional module	Representative evidence	Evidence status	Recommended interpretation
m6A-dependent RNA stabilization	HNRNPA2B1 binding and stabilization of selected m6A-modified lncRNAs or mRNAs in specific cancer models	Relatively supported	A transcript-specific mechanism validated in selected contexts
Selective nuclear export	Interaction with export-related machinery and enhanced export of selected m6A-tagged transcripts	Context-specific evidence	A possible export-related function, not a universal mechanism
m6A-independent translation regulation	Cytoplasmic HNRNPA2B1 interaction with PABPC1/eIF4F under H. pylori-associated conditions	Model-specific evidence	A non-canonical function requiring validation in other cancers
EV RNA sorting	HNRNPA2B1-associated miRNA sorting into extracellular vesicles	Supported in selected EV models	A potential intercellular RNA communication module
Immune–metabolic adaptation	Associations with lactate accumulation, ferroptosis resistance, and immune-cell infiltration	Partially supported/hypothesis-generating	A phenotype-level association requiring direct causal validation
Therapy resistance	Links to chemotherapy, radiotherapy, endocrine therapy, or targeted therapy resistance in selected studies	Variable evidence	Treatment- and cancer-type-specific, not a universal resistance mechanism

Taken together, current evidence supports the view that HNRNPA2B1 is a multifunctional and context-responsive regulator involved in several dimensions of post-transcriptional control in cancer. However, the concept of HNRNPA2B1 as an RNA fate hub should presently be regarded as an emerging and evidence-guided framework rather than a fully unified or universally established model. Recognizing these boundaries is essential not only for accurate interpretation of the current literature, but also for identifying the most meaningful directions for future mechanistic and translational investigation.

## 10 Translational potential, challenges, and future priorities

### 10.1 Biological feasibility and context-specific dependency

A central challenge is determining whether HNRNPA2B1 represents a true cancer dependency or a context-dependent modifier of malignant phenotypes. Because HNRNPA2B1 participates in fundamental RNA metabolic processes, its expression or activity alone is unlikely to be sufficient for therapeutic prioritization. Future studies should identify cancer types, molecular subgroups, or treatment states in which HNRNPA2B1 perturbation produces selective tumor vulnerability. Such dependency may be more likely in tumors with specific m6A-marked RNA programs, stress-induced cytoplasmic relocalization, EV-associated RNA communication, ferroptosis suppression, or therapy-induced adaptive RNA regulation. However, these possibilities require validation using genetic perturbation, rescue experiments, patient-derived models, and *in vivo* systems.

### 10.2 Druggability and selectivity of HNRNPA2B1-centered pathways

Direct pharmacological targeting of HNRNPA2B1 is likely to be challenging because RNA-binding proteins often lack conventional enzymatic pockets and interact with multiple RNA substrates and protein partners. Non-selective inhibition of HNRNPA2B1 may disrupt basal RNA processing in normal cells, raising concerns about therapeutic selectivity. Therefore, rather than proposing broad HNRNPA2B1 inhibition, future strategies may need to focus on more selective intervention points, such as disrupting disease-relevant RNA–protein interactions, modulating context-specific post-translational modifications, or targeting downstream vulnerabilities created by HNRNPA2B1-associated RNA programs. Even these approaches remain largely experimental and require rigorous assessment of specificity, off-target effects, and functional consequences in normal tissues.

### 10.3 Toxicity and therapeutic window

The potential toxicity of targeting HNRNPA2B1-centered pathways requires careful consideration. As a multifunctional RNA-binding protein, HNRNPA2B1 may contribute to RNA processing, transport, translation-related regulation, and extracellular RNA sorting in both malignant and non-malignant cells. Systemic inhibition could therefore affect rapidly proliferating normal tissues, immune cells, hematopoietic compartments, or stress-responsive epithelial cells. A therapeutic window may exist only if tumor cells exhibit disproportionate dependence on a specific HNRNPA2B1-associated module that is less essential in normal tissues. Establishing this window will require comparative studies in tumor cells, normal matched cells, organoids, and animal models.

### 10.4 Evidence-based translational priorities

Several priorities should guide future translational studies. First, direct HNRNPA2B1 substrates should be identified using integrated CLIP-seq, MeRIP-seq, RNA stability assays, subcellular RNA profiling, polysome profiling, and EV RNA analysis. Second, causal links between HNRNPA2B1-regulated RNA events and clinically relevant phenotypes should be tested using rescue experiments and patient-derived models. Third, biomarker strategies should move beyond total HNRNPA2B1 expression and incorporate functional readouts such as subcellular localization, RNA-binding signatures, m6A-marked target transcripts, EV RNA cargo, and therapy-induced RNA regulatory states. Finally, combination strategies should be proposed only when a specific dependency is experimentally validated, such as ferroptosis-related vulnerability in radioresistant tumors or immune–metabolic vulnerability in tumors with defined HNRNPA2B1-associated metabolic phenotypes.

In summary, HNRNPA2B1-associated pathways may have translational relevance, but their therapeutic exploitation remains at an early stage. The immediate priority is not to expand the list of hypothetical interventions, but to define where HNRNPA2B1 is mechanistically required, whether its functions are druggable with sufficient selectivity, and how potential toxicity can be minimized.

## 11 Conclusions

HNRNPA2B1 is emerging as a context-dependent regulator of selected RNA fate processes in cancer. Current evidence supports its involvement in several distinct RNA regulatory modules, including m6A-dependent RNA stabilization, selective nuclear export, cytoplasmic translation regulation, and EV-associated RNA sorting. These modules may contribute to cancer-associated phenotypes such as metabolic adaptation, immune remodeling, ferroptosis regulation, and therapy response. However, most mechanisms have been described in different cancer types, stimuli, or experimental systems, and should not be interpreted as components of a single universal HNRNPA2B1-centered cascade. Future studies should move from phenotype-level association toward integrated mechanistic validation. Approaches combining CLIP-seq, MeRIP-seq, subcellular RNA profiling, polysome profiling, EV RNA analysis, genetic perturbation, and functional rescue experiments will be required to define direct RNA substrates, mechanistic hierarchy, cancer-type specificity, and therapeutic relevance. A more evidence-stratified understanding of HNRNPA2B1 will help determine whether this RNA-binding protein can be translated from an emerging regulatory concept into a clinically actionable biomarker or therapeutic vulnerability.
